# Double thermoelectric power factor of a 2D electron system

**DOI:** 10.1038/s41467-018-04660-4

**Published:** 2018-06-20

**Authors:** Yuqiao Zhang, Bin Feng, Hiroyuki Hayashi, Cheng-Ping Chang, Yu-Miin Sheu, Isao Tanaka, Yuichi Ikuhara, Hiromichi Ohta

**Affiliations:** 1 Graduate School of Information Science and Technology, Hokkaido University, N14W9, Kita, Sapporo, 060−0814 Japan; 2 Institute of Engineering Innovation, The University of Tokyo, 2−11−16 Yayoi, Bunkyo, Tokyo, 113−8656 Japan; 3 Department of Materials Science and Engineering, Kyoto University, Yoshida-Honmachi, Sakyo, Kyoto, 606−8501 Japan; 4 Department of Electrophysics, National Chiao Tung University, 1001, University Rd, Hsinchu, 30010 Taiwan; 5 Center for Emergent Functional Matter Science, National Chiao Tung University, Hsinchu, 30010 Taiwan; 6 Research Institute for Electronic Science, Hokkaido University, N20W10, Kita, Sapporo, 001−0020 Japan

## Abstract

Two-dimensional electron systems have attracted attention as thermoelectric materials, which can directly convert waste heat into electricity. It has been theoretically predicted that thermoelectric power factor can be largely enhanced when the two-dimensional electron layer is far narrower than the de Broglie wavelength. Although many studies have been made, the effectiveness has not been experimentally clarified thus far. Here we experimentally clarify that an enhanced two-dimensionality is efficient to enhance thermoelectric power factor. We fabricated superlattices of [*N* unit cell SrTi_1−*x*_Nb_*x*_O_3_|11 unit cell SrTiO_3_]_10_—there are two different de Broglie wavelength in the SrTi_1−*x*_Nb_*x*_O_3_ system. The maximum power factor of the superlattice composed of the longer de Broglie wavelength SrTi_1−*x*_Nb_*x*_O_3_ exceeded ∼5 mW m^−1^ K^−2^, which doubles the value of optimized bulk SrTi_1−*x*_Nb_*x*_O_3_. The present approach—use of longer de Broglie wavelength—is epoch-making and is fruitful to design good thermoelectric materials showing high power factor.

## Abstract (web summary)

The accelerated growth of thermoelectric technology that efficiently converts waste heat to electricity necessitates the development of high-performance materials. Here, the authors experimentally demonstrate a 2D electron system with enhanced two-dimensionality and thermoelectric power factor.

## Introduction

Currently, more than 60% of the energy produced from fossil fuels is lost as waste heat. Thermoelectric energy conversion, which is the process where waste heat is transformed into electricity by the Seebeck effect, is attracting attention as a potential energy harvesting technology^1–4^. The performance of thermoelectric materials is generally evaluated in terms of a dimensionless figure of merit,

1 $$ZT = S^2 \cdot \sigma \cdot T \cdot \kappa ^{ - 1},$$

where *Z* is the figure of merit, *T* is the absolute temperature, *S* is the thermopower (Seebeck coefficient), *σ* is the electrical conductivity, and *κ* is the sum of the electronic (*κ*_ele_) and lattice thermal conductivities (*κ*_lat_) of a thermoelectric material.

There are two strategies to improve *ZT* of a thermoelectric material. One is to reduce *κ*_lat_. Recently, state-of-the-art nanostructuring techniques have reduced *κ*_lat_ significantly through phonon scattering by nanosized structural defects^5–8^. Such techniques have realized high-performance thermoelectric materials with a large *ZT* of 1.5−2. The other strategy is an enhancement of the product *S*^2^∙*σ*, which is called power factor (PF). However, it is extremely difficult to enhance PF due to the trade-off relationship between *S* and the carrier concentration (*n*). Therefore, PF has a local maximum value in three-dimensional (3D) bulk systems.

In a two-dimensional electron system (2DES) such as metal/insulator superlattices, electron carriers are confined within a thin layer (thickness thinner than the de Broglie wavelength, *λ*_D_). 2DES is an efficient strategy to achieve an enhanced PF. The effectiveness of 2DES was theoretically predicted by Hicks and Dresselhaus^9^; 2DES in extremely narrow layers exhibits an enhanced *S* without reducing *σ* because the density of states (DOS) near the bottom of the conduction band increases as the 2DES layer thickness decreases. These layers are narrower than the *λ*_D_,

2 $$\lambda _D = \frac{h}{{\sqrt {3 \cdot m^\ast \cdot k_{\mathrm{B}} \cdot T} }},$$

where *h*, m*, and *k*_B_ are Planck’s constant, effective mass of conductive electron or hole, and Boltzmann constant, respectively^9–13^.

Many experimental studies have been made to clarify the effectiveness of 2DES to enhance PF using PbTe/Pb_1−*x*_Eu_*x*_Te multiple-quantum-well^10^, electron-doped SrTiO_3_-based superlattices^14,15^, SiGe-based superlattices^16,17^, and Bi_2_Te_3_-based superlattices^18^. These 2DES layers showed enhanced *S*. However, total enhancement of PF was very small because of the insulator layer thickness. Thus, the effectiveness of 2DES has not been experimentally clarified thus far.

Here we experimentally clarify that an enhanced two-dimensionality is efficient to improve thermoelectric PF. We fabricated superlattices of [*N* unit cell SrTi_1−*x*_Nb_*x*_O_3_|11 unit cell SrTiO_3_]_10_—there are two different de Broglie wavelength in the SrTi_1−*x*_Nb_*x*_O_3_ system. The maximum PF of the superlattice composed of the longer de Broglie wavelength SrTi_1−*x*_Nb_*x*_O_3_ exceeded ~5 mW m^−1^ K^−2^, which doubles the value of optimized bulk SrTi_1−*x*_Nb_*x*_O_3_. The present approach—use of longer de Broglie wavelength—is epoch-making and is fruitful to design good thermoelectric materials showing high PF.

## Results

### Hypothesis

**Fig. 1:**
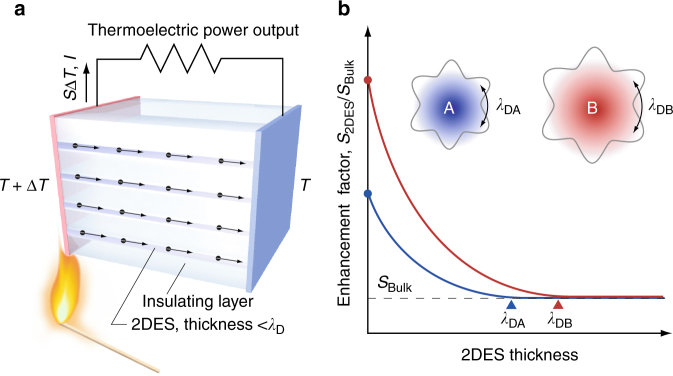
Thermoelectric effect of a 2D electron system. **a** Schematic illustration of thermoelectric Seebeck effect in a 2DES. A thermoelectric power output (*S*·Δ*T*·*I*) can be obtained when Δ*T* is introduced. **b** The hypothesis that a 2DES with longer de Broglie wavelength (*λ*_D_) shows a larger enhanced factor of thermopower

**Fig. 2:**
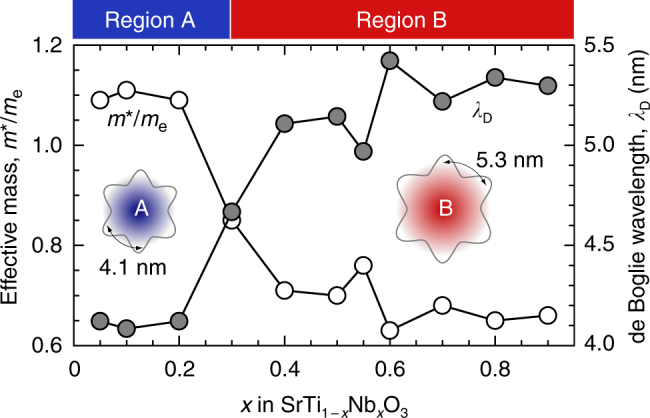
SrTiO_3_–SrNbO_3_ solid solution: a model system having two different *λ*_D_. *x*-dependent effective mass (m*/*m*_e_, white symbols) and *λ*_D_ (gray symbols) for SrTi_1 − *x*_Nb_*x*_O_3_ solid solutions. m*/*m*_e_ exerts a decreasing tendency with *x*, resulting in an increased *λ*_D_. Sharp changes in both m*/*m*_e_ and *λ*_D_ are detected around *x* = 0.3 due to the conduction band transition from Ti 3*d* to Nb 4*d*. The properties of SrTi_1 − *x*_Nb_*x*_O_3_ solid solutions can be divided into two regions based on the conduction bands (Ti 3*d* → region A and Nb 4*d* → region B). Inset: schematic illustrations of conduction electrons at regions A and B. At region B, *λ*_D_ is ∼5.3 nm, while it is ∼4.1 nm at region A

In order to enhance total PF of 2DES, two-dimensionality should be enhanced. Use of longer *λ*_D_ should be effective if the electron carriers are confined within a defined thickness layer (Fig. 1). Very recently, we observed a steep decrease in m*/*m*_e_ at *x* ~ 0.3 in SrTiO_3_–SrNbO_3_ solid solution system, SrTi_1−*x*_Nb_*x*_O_3_ (*x* is ranging from 0.05 to 0.9; Fig. 2)^19^. The ratio *x* of SrTi_1−*x*_Nb_*x*_O_3_ can be divided into two regions, region A (*x* is <0.3) and region B (*x* is >0.3). The origin of the two regions is most likely due to the difference in the overlap population between the Ti 3*d* and Nb 4*d* orbitals (*r*_Ti3*d*_ is 48.9 pm and *r*_Nb4*d*_ is 74.7 pm)^20^. We calculated *λ*_D_ values of SrTi_1−*x*_Nb_*x*_O_3_ using the Eq. (2). The *λ*_D_ value in region B is ~5.3 nm, which is 27% longer than that in region A (~4.1 nm). One can expect that *S*-enhancement factor in region B is much higher than that in region A because of higher two-dimensionality. Therefore, we hypothesized that SrTi_1−*x*_Nb_*x*_O_3_-based 2DES can be used to clarify the effectiveness of 2DES to enhance PF experimentally.

We fabricated [*N* uc SrTi_1−*x*_Nb_*x*_O_3_|11 uc SrTiO_3_]_10_ superlattices (*N* is ranging from 1 to 12, *x* is ranging from 0.2 to 0.9) by a pulsed laser deposition (PLD) technique on insulating (001) LaAlO_3_ (pseudo-cubic perovskite, the lattice parameter, *a* is 3.79 Å) single-crystal substrates using dense ceramic disks of a SrTiO_3_–SrNbO_3_ mixture and SrTiO_3_ single crystal as the targets. The thicknesses of different layers were monitored in situ using the intensity oscillation of the reflection high-energy electron diffraction (RHEED) spots. (See Experimental Section.) High-resolution X-ray diffraction (XRD) measurements revealed that the resultant superlattices were heteroepitaxially grown on (001) LaAlO_3_ with cube-on-cube epitaxial relationship with superlattice structure. Atomically smooth surfaces with stepped and terraced structure were observed by an atomic force microscopy (AFM).

### Microstructure and electronic structure

**Fig. 3:**
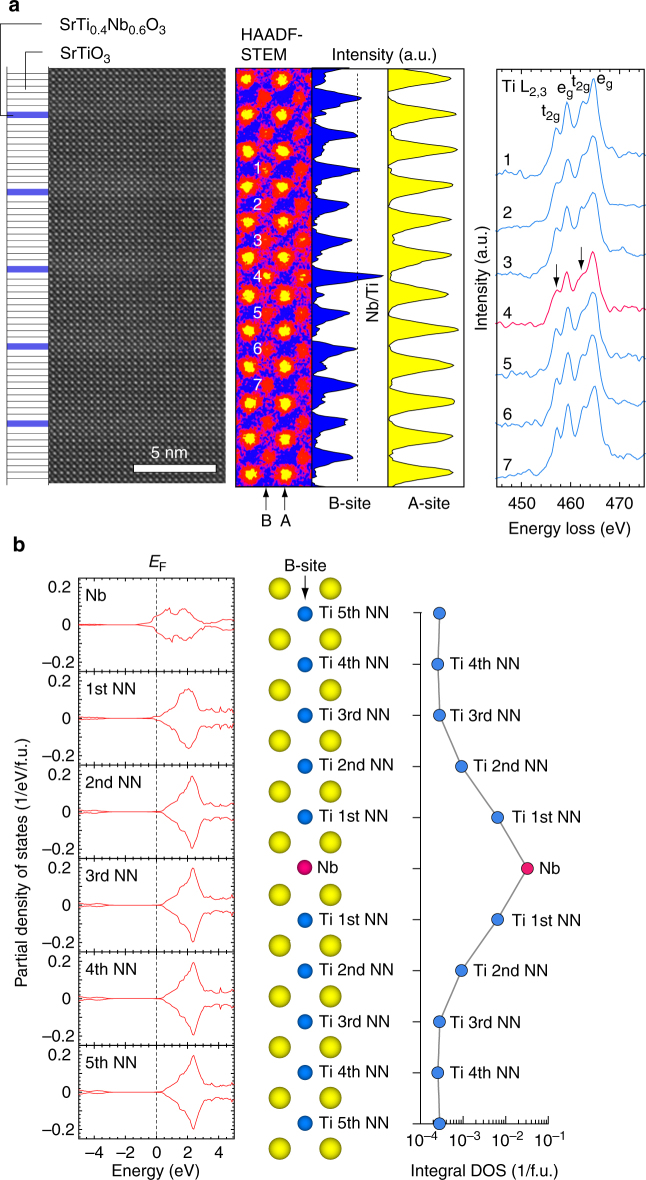
Experimental and theoretical analyses of the 2DES. **a** Cross-sectional HAADF-STEM image of the [1 uc SrTi_0.4_Nb_0.6_O_3_|11 uc SrTiO_3_]_10_ superlattice. Layer stacking sequence is also shown. Rather bright bands are seen near each SrTi_0.4_Nb_0.6_O_3_ layer. In the magnified image, the #4 atom in the B-site column is brighter than the nearby atoms, whereas no obvious difference is observed in the A-site column. EELS spectrum of #4 is broader than that of nearby atoms, indicating the coexistence of Ti^4+^/Ti^3+^ in the SrTi_0.4_Nb_0.6_O_3_ layers. **b** The calculated partial DOS of Nb 4*d* or Ti 3*d* in the [1 uc SrNbO_3_|10 uc SrTiO_3_] superlattice. The Fermi energy (*E*_F_) is located on the higher-energy side of the conduction band minimum for the first and second nearest neighbor (Ti first NN and Ti second NN). SrTiO_3_ layers together with 1 uc SrNbO_3_ layer (Nb) suggest that the electron carriers can seep from the SrTi_1 − *x*_Nb_*x*_O_3_ layers into the SrTiO_3_ layers

Figure 3a summarizes the atomic arrangements of the [1 uc SrTi_0.4_Nb_0.6_O_3_|11 uc SrTiO_3_]_10_ superlattice. Rather bright bands are observed near each SrTi_0.4_Nb_0.6_O_3_ layer in the Cs-corrected high-angle annular dark-field scanning transmission electron microscopy (HAADF-STEM) image. In the magnified image, the #4 atom in the B-site column is brighter than the nearby atoms. However, there is no obvious difference in the A-site column, indicating Nb substitution occurs for the #4 atom in the B-site column. The electron energy loss spectroscopy signal of #4 is broader than that of the nearby atoms, implying the coexistence of Ti^4+^/Ti^3+^ in the SrTi_0.4_Nb_0.6_O_3_ layers^21^. Therefore, in our superlattice fabrication, Nb ions are successfully confined into 1 uc of SrTi_0.4_Nb_0.6_O_3_ layers^22^.

In order to clarify the 2DES formation, the electronic band structures of the [1 uc SrNbO_3_|10 uc SrTiO_3_] superlattices were calculated based on the projector-augmented wave (PAW) method (Fig. 3b). The *E*_F_ is located on the higher-energy side of the conduction band minimum for the first and second nearest-neighbor SrTiO_3_ layers (Ti first NN and Ti second NN) together with the 1 uc SrNbO_3_ layer (Nb). The electron carriers can seep from the SrNbO_3_ layers into the SrTiO_3_ layer. Delugas et al.^23^ have also predicted theoretically that for lower Nb substituted samples, it is much easier for the electrons, especially in the *d*_*xz*_ and *d*_*yz*_ bands, to spread out to the neighboring SrTiO_3_ layers, reducing the two-dimensionality. However, as the Nb content increases, the minimum thickness of the barrier layer may be reduced to 5 uc in the SrNbO_3_ case. There is no doubt that the electron diffusion cannot be removed thoroughly in superlattice structure, but diffusion effects can be effectively suppressed by the high Nb substitution. From the band calculation, 2DES in our work is mainly confined to the 1 uc SrTi_1−*x*_Nb_*x*_O_3_ layers and should contribute to the *S* enhancement.

In order to further confirm the superlattice structure, we measured the *κ* of the [1 uc SrTi_0.4_Nb_0.6_O_3_|11 uc SrTiO_3_]_10_ superlattice along the cross-plane direction by time-domain thermal reflectance (TDTR) method. The total *κ* could be suppressed to ~3.3 W m^−1^ K^−1^, similar to the minimum value of CaTiO_3_/SrTiO_3_-based superlattices (*κ* ~ 3.2 W m^−1^ K^−1^) reported by Ravichandran et al.^24^. From these results, we judged that our [*N* uc SrTi_1−*x*_Nb_*x*_O_3_|11 uc SrTiO_3_]_10_ superlattices (*N* is ranging from 1 to 12, *x* is ranging from 0.2 to 0.9) are appropriate for us to clarify the effectiveness of 2DES to enhance PF.

### Thermoelectric properties

**Fig. 4:**
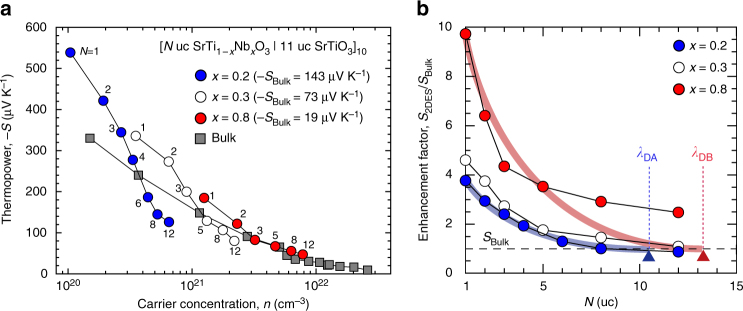
Two-dimensionality of 2DES: a key to enhance thermopower. **a** Plots of thermopower of the 2DESs, [*N* uc SrTi_1 − *x*_Nb_*x*_O_3_|11 uc SrTiO_3_]_10_ superlattices (*x* = 0.2, 0.3, and 0.8), versus the carrier concentration (*n*). Compared to bulk values (gray squares), all the 2DESs show an enhanced −*S* as *N* is reduced under 3 uc. **b** Enhancement factors in –*S* (*S*_2DES_/*S*_Bulk_) for three sets of 2DESs. For *x* = 0.2 and 0.3 2DESs, the highest *S*_2DES_/*S*_Bulk_ values are obtained at *N* = 1, which are 4 and 5, respectively, while that of *x* = 0.8 can reach 10

The electrical conductivity (*σ*), carrier concentration (*n*), and Hall mobility (*μ*_Hall_) of the superlattices were measured at room temperature by a conventional d.c. four-probe method with a van der Pauw geometry. *S* was measured at room temperature by creating a temperature difference (Δ*T*) of ~4 K across the film using two Peltier devices. Figure 4a summarizes the *n*-dependent *S* of [*N* uc SrTi_1−*x*_Nb_*x*_O_3_|11 uc SrTiO_3_]_10_ superlattices (*N* is ranging from 1 to 12, *x* = 0.2, 0.3, and 0.8) along with bulk (~100-nm-thick SrTi_1−*x*_Nb_*x*_O_3_ films, *x* = 0.2, 0.3, and 0.8, respectively) values for comparison. The bulk *S* for *x* = 0.2 was −143 μV K^−1^, *x* = 0.3 was −73 μV K^−1^, and *x* = 0.8 was −19 μV K^−1^. The *n* value was measured based on the total thickness of the 2DES, which includes the insulating SrTiO_3_ layers. All the 2DES samples show enhanced thermopower (−*S*) with a reduced *N*. Compared to the bulk samples at a similar *n*, a much higher −*S* is observed in superlattices as *N* is reduced below 3 uc.

To confirm the increasing two-dimensionality with *x*, the *S*-enhancement factors (*S*_2DES_/*S*_Bulk_) were plotted versus the *N* values (Fig. 4b). For 2DES with *x* = 0.2 and 0.3, the highest *S*_2DES_/*S*_Bulk_ values are around 4 and 5, respectively, whereas that for the *x* = 0.8 counterpart is ~10. As hypothesized, the enhanced *S*_2DES_/*S*_Bulk_ should stem from the increasing *λ*_D_ with *x*. In our experiment, *S*_2DES_/*S*_Bulk_ for the *x* = 0.2 and 0.3 2DESs are saturated around 11 uc, which is consistent with *λ*_D_ in region A (~4.2 nm indicated by dashed line *λ*_DA_). As *λ*_D_ increases in region B, the saturation position for the *x* = 0.8 2DES has a thickness larger than the *λ*_D_ (~5.2 nm indicated by dashed line *λ*_DB_). As a result, a significantly enhanced two-dimensionality is achieved in the *x* > 0.3 region B, which fits well with our hypothesis and suggests that region B has the potential to further enhance the thermoelectric PF.

**Fig. 5:**
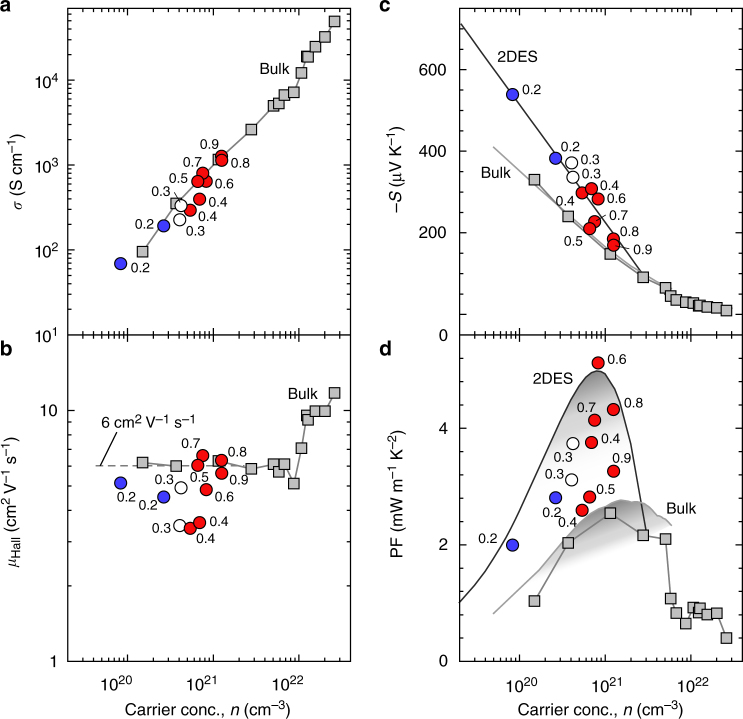
Double enhancement of the thermoelectric power factor in a 2DES. Carrier concentration dependences of **a** electrical conductivity (*σ*), **b** Hall mobility (*μ*_Hall_), **c** thermopower (−*S*), and **d** power factor [PF (*S*^2^·*σ*)] of [1 uc SrTi_1 − *x*_Nb_*x*_O_3_ | 11 uc SrTiO_3_]_10_ 2DESs (*x* is ranging from 0.2 to 0.9) at room temperature. Similar to the trends in the bulk values, *σ* increases almost linearly with *n*. *μ*_Hall_ for lower *x* samples (*x* ≤ 0.5) fluctuates around 3–5 cm^2^ V^−1^ s^−1^, while that for higher *x* ones (*x* ≥ 0.6) is ~6 cm^2^ V^−1^ s^−1^. Slope of –*S* versus log *n* for bulk SrTi_1 − *x*_Nb_*x*_O_3_ is –198 μV K^−1^, which is ∼1.5 times lower than –300 μV K^−1^ for the 2DESs. Double enhancement of PF is seen in *x* = 0.6 (5.1 mW m^−1^ K^−2^ at *n* ∼ 8 × 10^20^ cm^−3^). Since the PF values are scattered due to the rather large distribution of *μ*_Hall_ (3–6 cm^2^ V^−1^ s^−1^), we calculated PFs using the relationship between *S* and *n* (c) at constant *μ*_Hall_ (6 cm^2^ V^−1^ s^−1^). The optimized PF of the 2DES should be ~5 mW m^−1^ K^−2^ at *n* ∼ 8 × 10^20^ cm^−3^, which doubles that of bulk SrTi_1 − *x*_Nb_*x*_O_3_ (PF ∼ 2.5 mW m^−1^ K^−2^ at *n* ∼ 2 × 10^21^ cm^−3^)

Based on the conclusions above, we have enhanced the thermoelectric PF in [1 uc SrTi_1−*x*_Nb_*x*_O_3_|11 uc SrTiO_3_]_10_ superlattices by adjusting *x* between 0.2 and 0.9. Figure 5 summarizes the *n* dependences of the thermoelectric properties of [1 uc SrTi_1−*x*_Nb_*x*_O_3_|11 uc SrTiO_3_]_10_ superlattices at room temperature along with the reported bulk values for comparison^19^. Following the bulk values, *σ* increases almost linearly with *n* (Fig. 5a), indicating that *n* dominates *σ*. In the SrTi_1−*x*_Nb_*x*_O_3_ system, carriers are mostly due to Nb substitution. The high *n* also induces a highly Nb substituted region with a superiority in *σ*. However, *σ* for the superlattices remains lower than the bulk value due to the coexistence of 11 uc SrTiO_3_ insulating layers.

*μ*_Hall_ for lower *x* of 2DESs (*x* ≤ 0.5) fluctuates around 3–5 cm^2^ V^−1^ s^−1^, while for higher *x* 2DESs (*x* ≥ 0.6) values are ~6 cm^2^ V^−1^ s^−1^ (Fig. 5b). Usually, *μ*_Hall_ is controlled by the conduction band of materials along with the effects of crystal defects such as impurities and grain boundaries. In the bulk samples, *μ*_Hall_ sharply increases due to the transition of the conduction band from Ti 3*d* to Nb 4*d* as *x* increases into the highly Nb substituted region^19^. This pattern is also observed in the superlattice counterparts. A higher *μ*_Hall_ (≥6 cm^2^ V^−1^ s^−1^) is observed in samples with *x* ≥ 0.6 than that for *x* ≤ 0.5 (3–5 cm^2^ V^−1^ s^−1^). Compared to the bulk samples, all the superlattices exert a much lower *μ*_Hall_, which may result from an insufficient crystal quality or electron diffusion into the pure SrTiO_3_ barrier layers. Regardless, a conduction band transition from Ti 3*d* to Nb 4*d* is recognized in our superlattice systems. Due to the high overlapping population of the Nb 4*d* orbital, a superior electron transport property is realized in higher *x* of 2DES.

Figure 5c plots the *S* values for all the superlattices versus *n* along with the reported bulk values^19^. The solid line depicts the overall tendency. In the diagram, the superlattices have a significantly enhanced –*S* compared to bulk samples at similar *n* values. As indicated by the solid lines, the experimental points for 2DES and bulk show different slopes of –300 and –200 μV K^−1^ per decade, respectively. The relationship between –*S* and *n*_eff_ can be expressed by Eq. (3)

3 $$- S = - k_{\mathrm{B}}{\mathrm{/}}e\cdot {\mathrm{ln}}\,10\cdot A\cdot \left( {{\mathrm{log}}\,{\it{n}} + B} \right),$$

where *k*_B_ is the Boltzmann constant and *e* is an electron charge. *A* and *B* are the parameters that depend on the type of materials and their electronic band structures. Bulk shows a 3D electronic band structure with a parabolic shaped DOS near *E*_F_, where the *A* value = 1 and the slope reflects a constant value of −*k*_B_/*e*·ln10 (−198 μV K^−1^). On the other hand, the slope of the 2DESs may reach −300 μV K^−1^ per decade, indicating that the *A* value = 1.5. Therefore, the 2DESs work well to enhance the *S* even for the whole superlattice, including SrTiO_3_ insulating layers.

Finally, we calculated PF of the [1 uc SrTi_1−*x*_Nb_*x*_O_3_|11 uc SrTiO_3_]_10_ superlattices (*x* is ranging from 0.2 to 0.9) using the observed *S* and *σ* values (Fig. 5d). PF is doubly enhanced for *x* = 0.6 (5.1 mW m^−1^ K^−2^ at *n* ~ 8 × 10^20^ cm^−3^). Since the PF values are scattered due to the rather large distribution of *μ*_Hall_ (3–6 cm^2^ V^−1^ s^−1^), we calculated PFs using the relationship between *S* and *n* (c) at constant *μ*_Hall_ (6 cm^2^ V^−1^ s^−1^). The optimized PF of the 2DES should be ~5 mW m^−1^ K^−2^ at *n* ~ 8 × 10^20^ cm^−3^, which doubles that of bulk SrTi_1−*x*_Nb_*x*_O_3_ (PF ~ 2.5 mW m^−1^ K^−2^ at *n* ~ 2 × 10^21^ cm^−3^).

## Discussion

The present 2DES, [1 uc SrTi_1−*x*_Nb_*x*_O_3_|11 uc SrTiO_3_]_10_ superlattices (*x* is ranging from 0.2 to 0.9), has several merits to enhance PF as compared with other 2DESs such as PbTe/Pb_1−*x*_Eu_*x*_Te multiple-quantum-well^10^, SiGe-based superlattices^16,17^, and Bi_2_Te_3_-based superlattices^18^, which are already commercialized thermoelectric materials. This is because SrTi_1−*x*_Nb_*x*_O_3_ can be deposited with 1 uc layer accuracy by PLD. Therefore, we can easily reduce the 2DEG thickness to ~0.4 nm (1 uc layer). Further, there are two different *λ*_D_ in SrTi_1−*x*_Nb_*x*_O_3_; ~4.1 nm in the low conducting region and ~5.3 nm in the high conducting region. For enhancing PF, both *S* and *σ* play important roles. The present research implies that high conducting region is effective to enhance the thermoelectric PF in the 2DES. Herein highly Nb substitution are revealed to have the coexistence of both a high electron transport (high *n* and *μ*_Hall_) and a high two-dimensionality (large *λ*_D_).

In summary, we have experimentally clarified that an enhanced two-dimensionality of 2DES is efficient to improve thermoelectric PF. We measured the thermoelectric properties of 2DESs [*N* uc SrTi_1−*x*_Nb_*x*_O_3_|11 uc SrTiO_3_]_10_ superlattices (*N* is ranging from 1 to 12, *x* is ranging from 0.2 to 0.9) because there are two different *λ*_D_ in this 2DES (*x* > 0.3: *λ*_D_~5.3 nm; *x* < 0.3: *λ*_D_~4.1 nm). The *S*-enhancement factor *S*_2DES_/*S*_Bulk_ of the 2DES (*N* = 1) for *x* > 0.3 were ~10, whereas those for *x* < 0.3 were 4–5. Maximum PF of the 2DES (*N* = 1, *x* = 0.6) exceeded ~5 mW m^−1^ K^−2^, which doubles the value of optimized bulk SrTi_1−*x*_Nb_*x*_O_3_ (PF ~ 2.5 mW m^−1^ K^−2^). The present 2DES approach—use of longer *λ*_D_—is epoch-making and is fruitful to design good thermoelectric materials showing high PF.

## Methods

### Fabrication and analyses of the 2DESs

A series of superlattices with the chemical formula of [*N* uc SrTi_1−*x*_Nb_*x*_O_3_ | 11 uc SrTiO_3_]_10_ (*N* is ranging from 1 to 12, *x* is ranging from 0.2 to 0.9) were fabricated by a PLD technique using dense ceramic disks of a SrTiO_3_–SrNbO_3_ mixture and a SrTiO_3_ single crystal as the targets. The substrate was insulating (001) LaAlO_3_ (pseudo-cubic perovskite, lattice parameter, *a* is 3.79 Å, the surface area: 1 cm × 1 cm). The growth conditions were precisely controlled; the substrate temperature was 900 °C, the oxygen pressure was ~10^−4^ Pa, and the laser fluence was ~1.2 J cm^−2^ per pulse. The thicknesses of different layers were monitored in situ using the intensity oscillation of the RHEED spots. Details of our PLD growth process of the superlattices are reported elsewhere^14,25^.

Crystallographic analyses of the resultant superlattices were performed by XRD (Cu Kα_1_, ATX-G, Rigaku Co.), AFM (Nanocute, Hitachi Hi-Tech), and STEM (200 keV, JEM-ARM 200CF, JEOL Co. Ltd). TEM samples were fabricated using a cryo ion slicer (IB-09060CIS, JEOL Co. Ltd). HAADF images were taken with the detection angle of 68–280 mrad. Electron energy loss spectra were acquired in STEM mode with the energy resolution of 0.8 eV.

### Measurements of the thermoelectric properties of the 2DESs

Electrical conductivity (*σ*), carrier concentration (*n*), and Hall mobility (*μ*_Hall_) were measured at room temperature by a conventional d.c. four-probe method with a van der Pauw geometry. *S* was measured at room temperature by creating a temperature difference (Δ*T*) of ~4 K across the film using two Peltier devices. (Two small thermocouples were used to monitor the actual temperatures of each end of a superlattice.) The thermo-electromotive force (Δ*V*) and Δ*T* were measured simultaneously, and the *S* values were obtained from the slope of the Δ*V*–Δ*T* plots (the correlation coefficient: >0.9999).

Cross-plane thermal conductivity (*κ*) was measured by TDTR (Picotherm Co.) method. Mode-locked fiber pulse lasers with 1550 and 775 nm wavelengths were used for heating and measuring, respectively. Both lasers are with the repetition frequency of 20 MHz and pulse duration of 0.4 ps. Before measurement, Mo film with a thickness of 100 nm was first deposited on the surface of the sample as the transducer. During measurement, time-dependent transient thermoreflectance phase signal of Mo transducer was measured, from which *κ* was further simulated. Time-domain thermoreflectance was measured based on amplified laser systems (5 kHz and ~200 fs centered at 1030 nm). Degenerate pump and probe photons were separated by the cross polarization, and a polarizing filter was employed before the lock-in detection. A mechanical delay stage was used for time scan up to 1.5 ns. Pump to probe intensity ratio was >15, and the size ratio was around 6.

### Energy band calculation of the 2DES

Band structure for the [1 uc SrNbO_3_|10 uc SrTiO_3_] superlattice was calculated based on the PAW method^26^, as implemented in the VASP code^27,28^. We adopted the Heyd–Scuseria–Ernzerhof hybrid functionals^29–31^ and a plane-wave cutoff energy of 550 eV. 6 × 6 × 6 and 6 × 6 × 2 *k*-point meshes were employed in the total-energy evaluations and geometry optimization for the perovskite unit cells of SrTiO_3_ and the superlattice cell, respectively. The in-plane lattice constant of the superlattice cell was fixed at the optimized value of SrTiO_3_ while the out-of-plane lattice constant and the atomic coordinates were fully relaxed.

### Data availability

The data that support the findings of this study are available from the corresponding authors upon reasonable request.

## Electronic supplementary material

Peer Review File
